# Supplementary figures and images for: Characterization of phenylalanine ammonia-lyase genes facilitating flavonoid biosynthesis from two species of medicinal plant Anoectochilus
Source: PeerJ. 2022 Jul 6;10:e13614. doi: 10.7717/peerj.13614 (PMC9270878; doi:10.7717/peerj.13614)

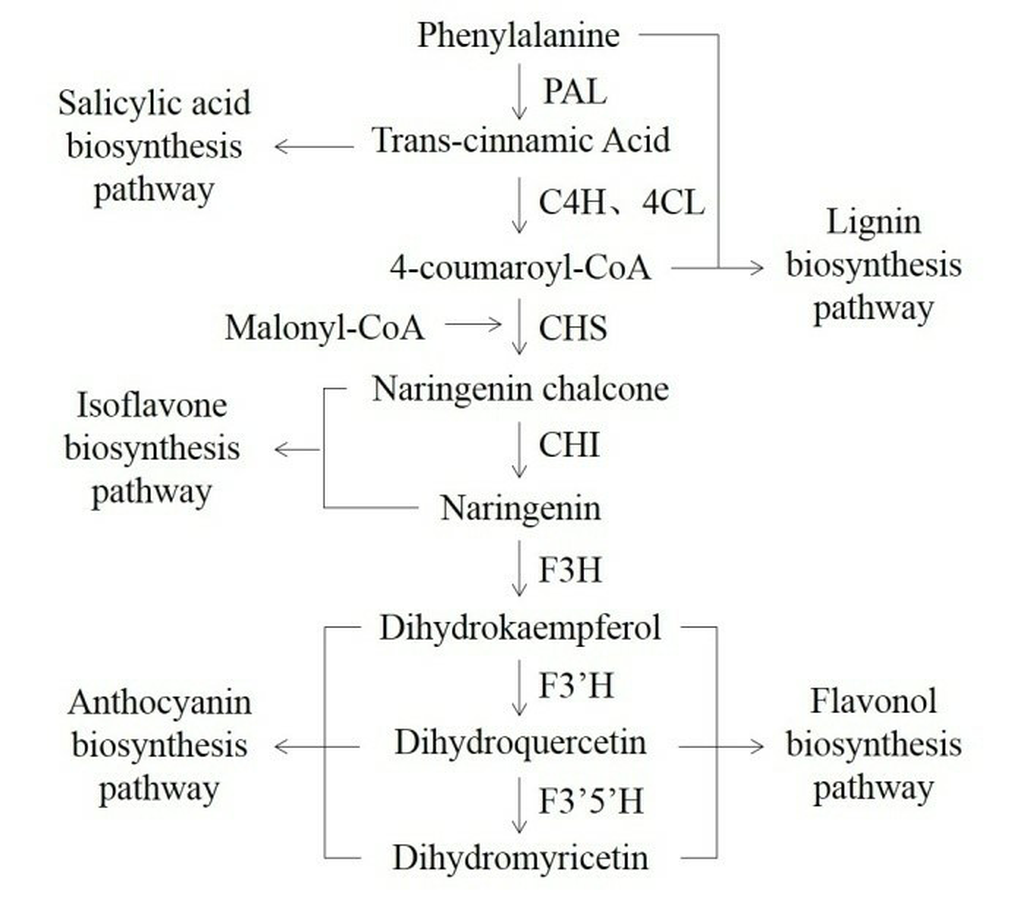

Supplement: Supplemental Information 1 [file peerj-10-13614-s001.png]

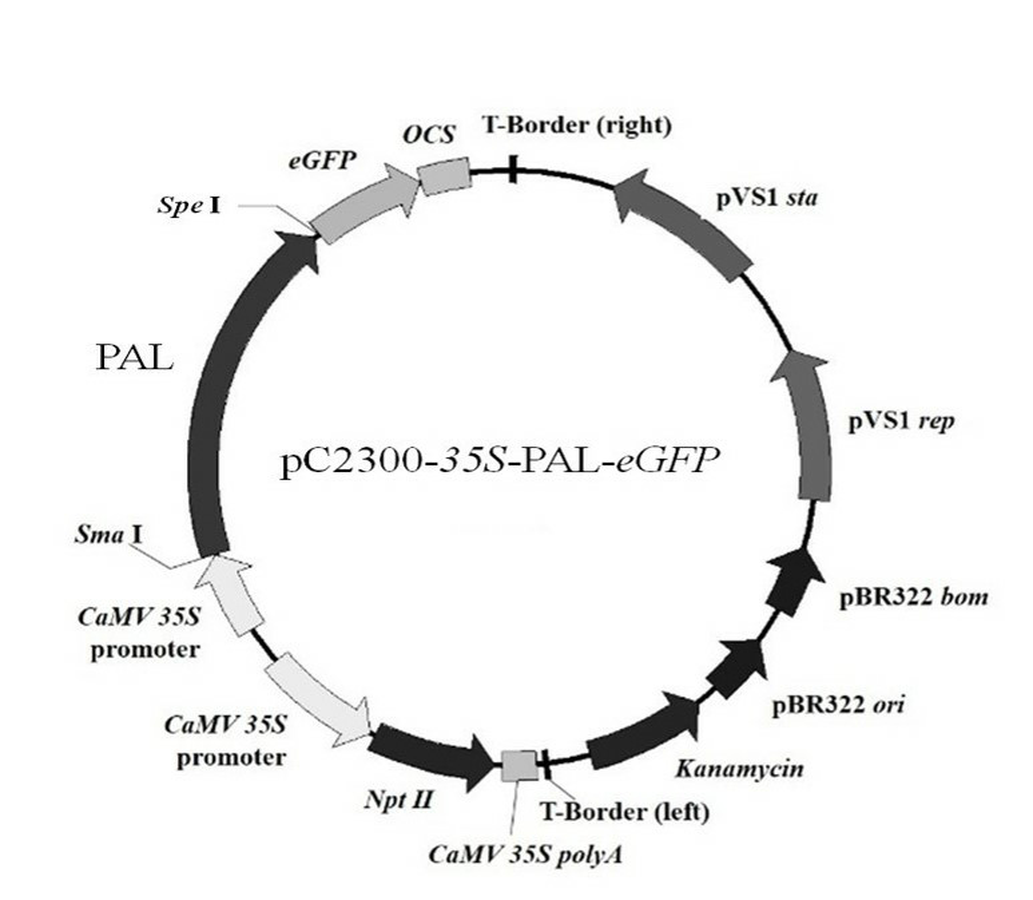

Supplement: Supplemental Information 2 [file peerj-10-13614-s002.png]

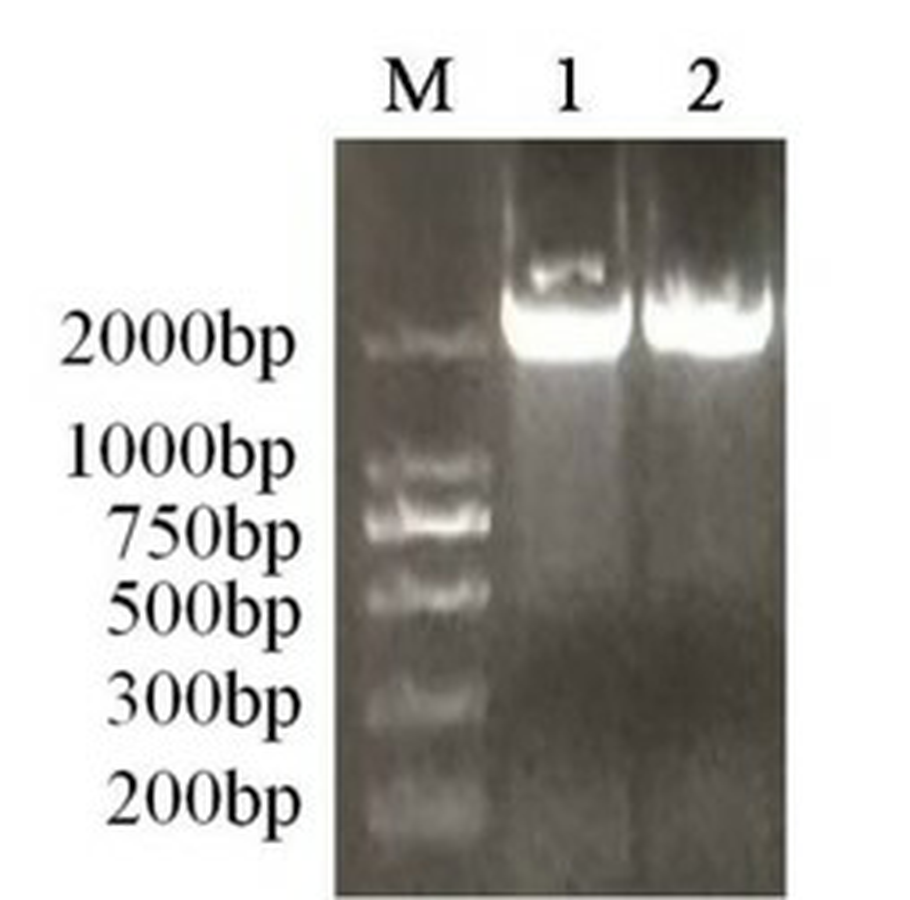

Supplement: Supplemental Information 3 — M represents marker; 1, 2 represents A. formosanus and A. roxburghi ai. [file peerj-10-13614-s003.png]

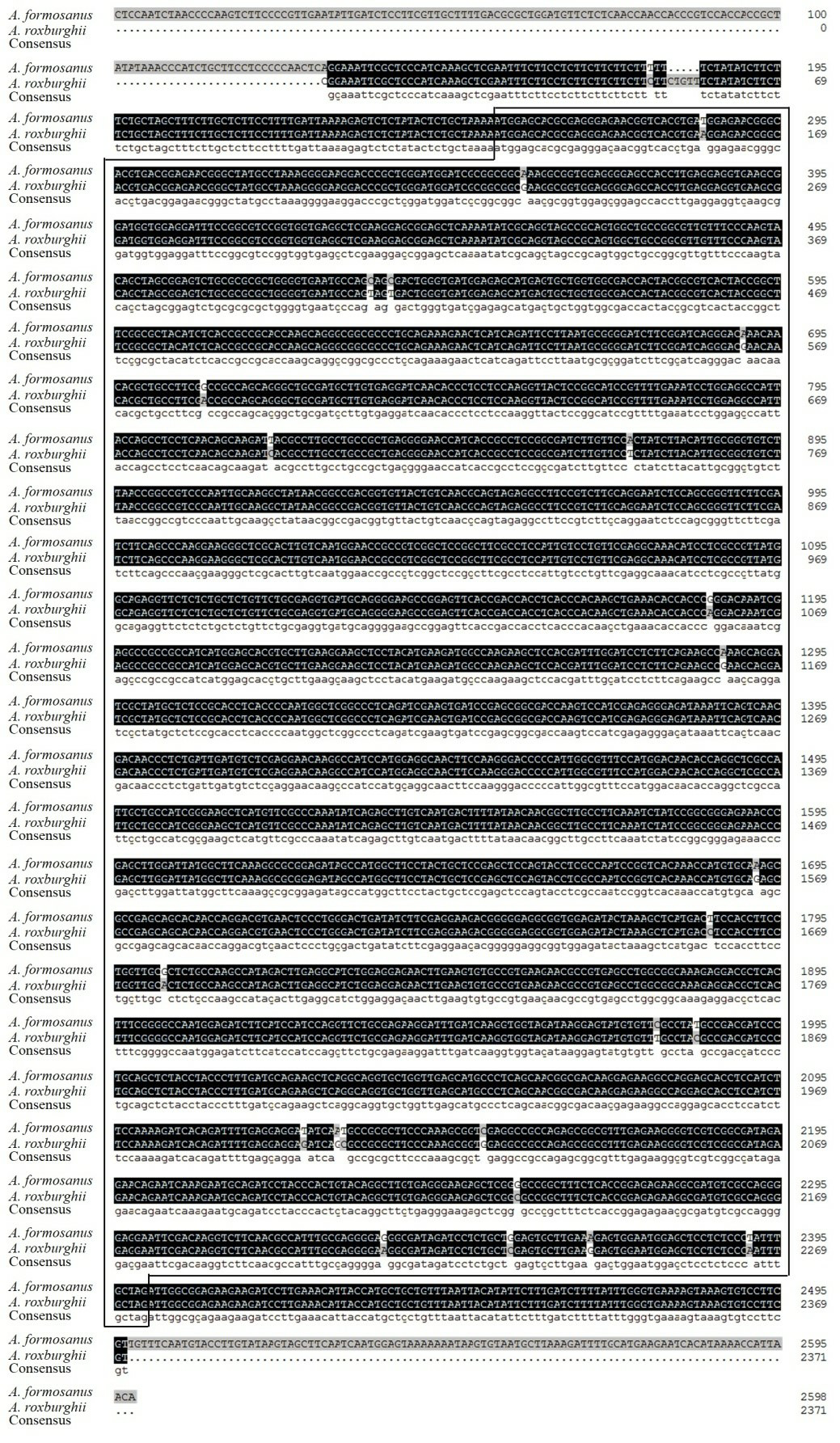

Supplement: Supplemental Information 4 — Identical and conserved bases are denoted by black (100%), gray (66.6%) and white (0%) backgrounds, respectively, and the box represent the ORF. [file peerj-10-13614-s004.png]

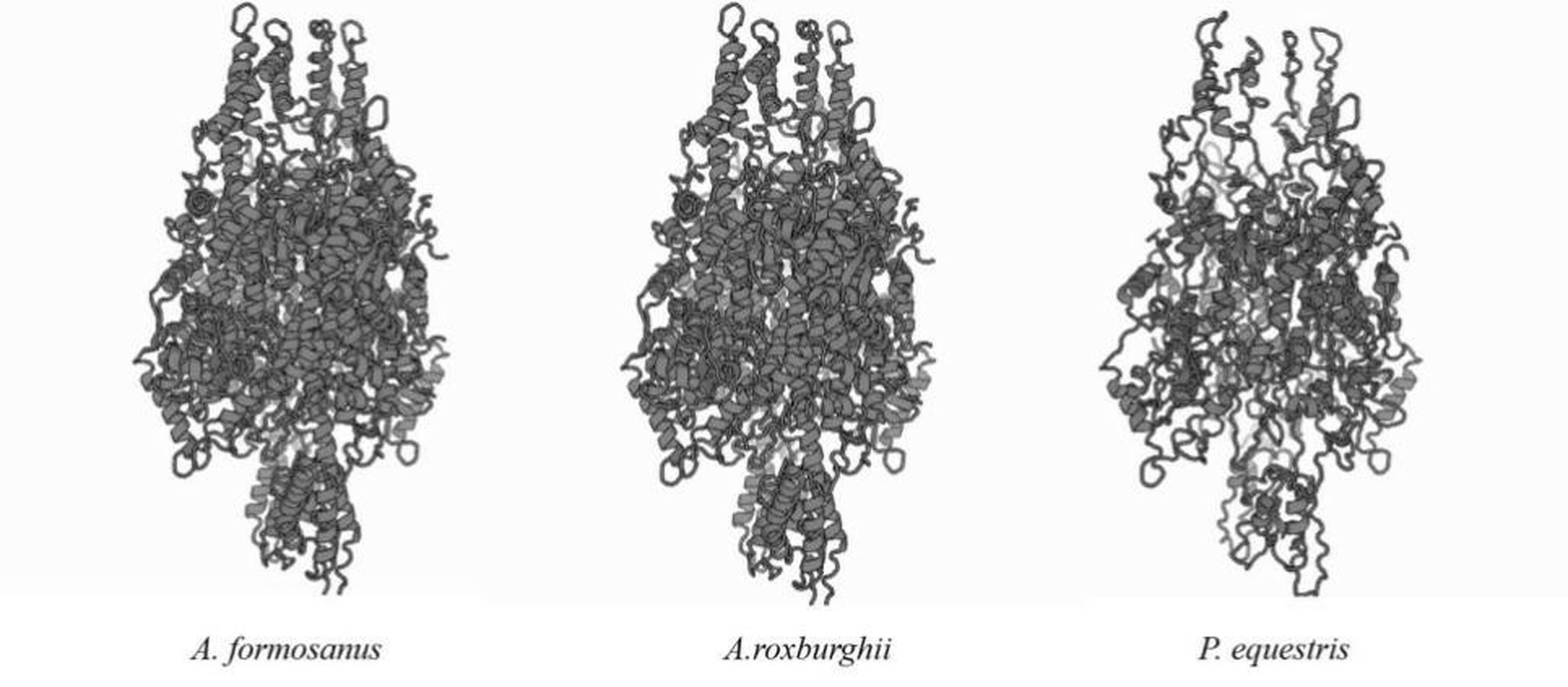

Supplement: Supplemental Information 5 [file peerj-10-13614-s005.png]

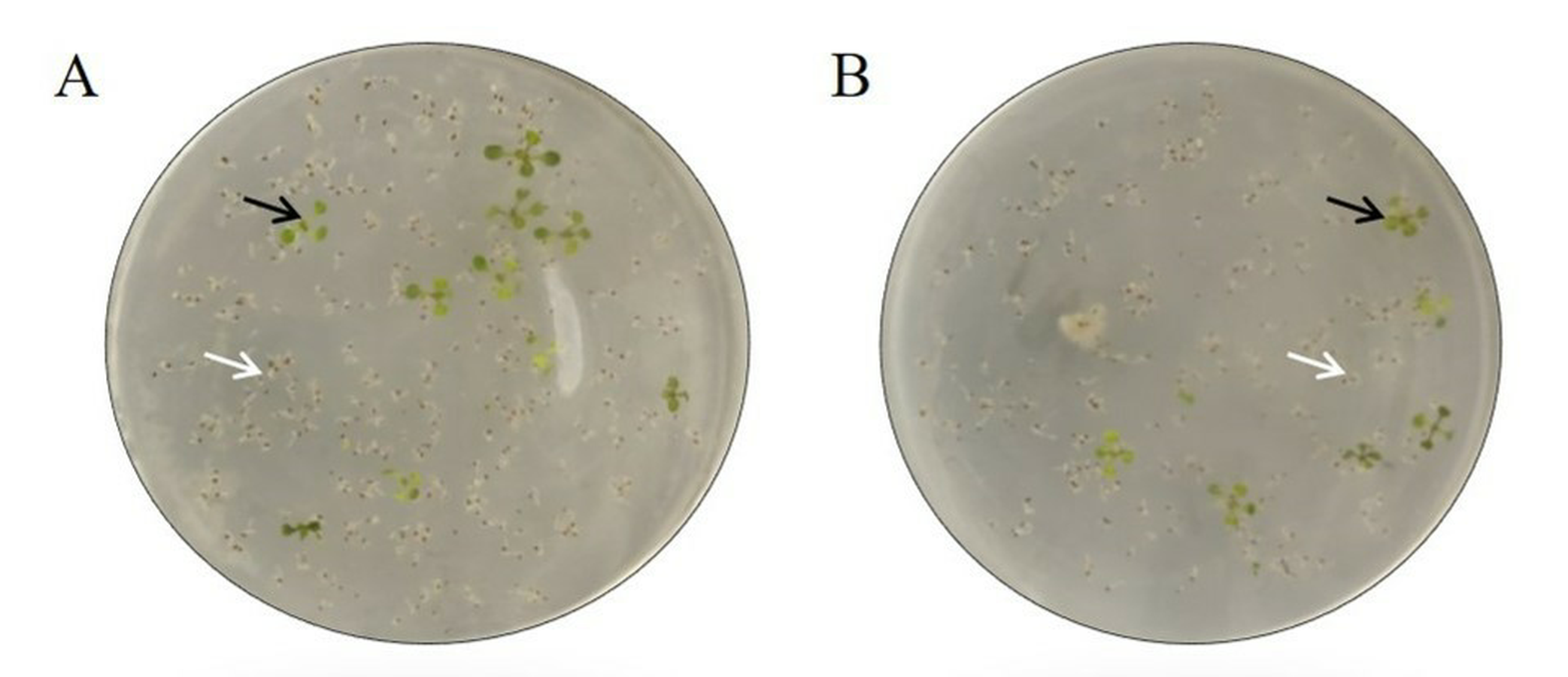

Supplement: Supplemental Information 6 — A and B represent A. formosanus and A.roxburghii, respectively; black arrows represent living Arabidopsis (overexpression); white arrows represent dead Arabidopsis (wild type). [file peerj-10-13614-s006.png]

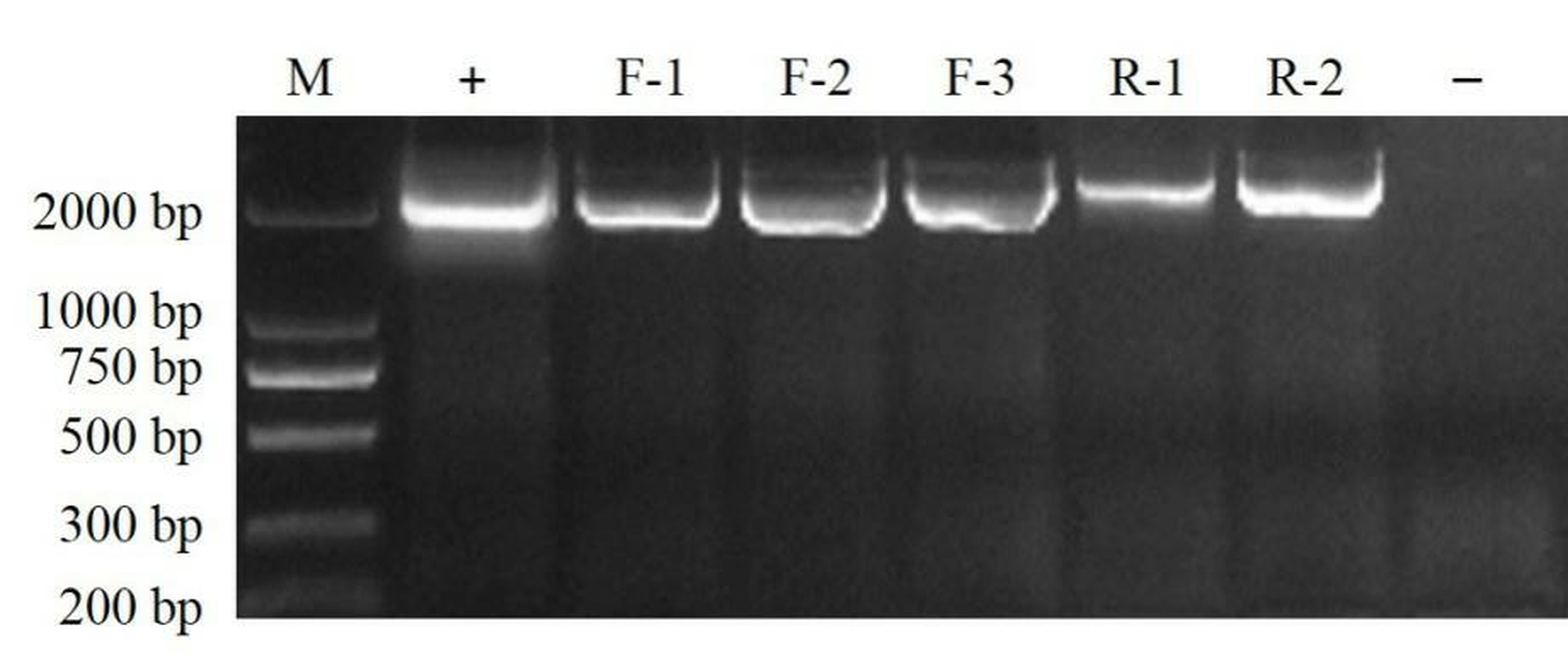

Supplement: Supplemental Information 7 — M: DNA molecular maker DL2000; +: Positive control (expression vecgtor pC2300-35S-PAL-eGFP); -: Negative control (untransformed line); F-1, F-2 and F-3: T3 line of gene PAL from A. formosanus; R-1 and R-2: T3 lines of gene PAL from A. roxburghii. [file peerj-10-13614-s007.png]
