# Supplementary material for: Characterization of phenylalanine ammonia-lyase genes facilitating flavonoid biosynthesis from two species of medicinal plant Anoectochilus
Source: PeerJ. 2022 Jul 6;10:e13614. doi: 10.7717/peerj.13614 (PMC9270878; doi:10.7717/peerj.13614)
Supplement: Supplemental Information 8 — AfPAL and ArPAL represent the sequence of phenylalanine ammonia-lyase gene from Anoectochilus formosanus and Anoectochilus roxburghii respectively; AfPAL and ArPAL represent the sequence of phenylalanine ammonia-lyase protein from Anoectochilus formosanus and Anoectochilus roxburghii respectively. [file peerj-10-13614-s008.docx]

>*AfPAL* [*Anoectochilus formosanus*] the sequence of phenylalanine ammonia-lyase gene from *Anoectochilus formosanus*

ATGGACCATGCTAGGGAGAACGGTCACGTGATGGAGAACGGGCACGTGACGGAGAACGGGCTATGCCTAAAGGGGAAGGACCCGCTGGGATGGATCGCGGCGGCAAAGGCGGTGGAGGGGAGCCACCTTGAGGAGGTGAAGCGGATGGTGGAGGATTTCCGGCGTCCGGTGGTGAGGCTCGAAGGAGCGGAGCTCAAAATATCGCAGGTAGCCGCAGTGGCTGCCGGCGTTGTTTCCCAAGTACAGCTAGCGGAGTCTGCGCGCGCTGGGGTGAATGCCAGCAGCGACTGGGTGATGGAGAGCATGAGTGCTGGTGGCGACCACTACGGCGTCACTACCGGCTTCGGCGCTACATCTCACCGCCGCACCAAGCAGGGCGGCGCCCTGCAGAAAGAACTCATCAGATTCCTTAATGCGGGGATCTTCGGATCAGGGACAAACAACACGCTGCCTTCGGCCGCCAGCAGGGCTGCGATGCTTGTGAGGATCAACACCCTCCTCCAAGGTTACTCCGGCATCCGTTTTGAAATCCTGGAGGCCATTACCAGCCTCCTCAACAGCAAGATTACGCCTTGCCTGCCGCTGAGGGGAACCATCACCGCCTCCGGCGATCTTGTTCCACTATCTTACATTGCGGGTGTCTTAACCGGCCGTCCCAATTGCAAGGCTATAACGGCCGACGGTGTTACTGTCAACGCAGTAGAGGCCTTCCGTCTTGCAGGAATCTCCAGCGGGTTCTTCGATCTTCAGCCCAAGGAAGGGCTCGCACTTGTCAATGGAACCGCCGTCGGCTCCGGCTTCGCCTCCATTGTCCTGTTCGAGGCAAACATCCTCGCCCTTATGGCAGAGGTTCTCTCTGCTCTGTTCTGCGAGGTGATGCAGGGGAAGCCGGAGTTCACCGACCACCTCACCCACAAGCTGAAACACCACCCGGGACAAATCGAGGCCGCCGCCATCATGGAGCACGTGCTTGAAGGAAGCTCCTACATGAAGATGGCCAAGAAGCTCCACGATTTGGATCCTCTTCAGAAGCCAAAGCAGGATCGCTATGCTCTCCGCACCTCACCCCAATGGCTCGGCCCTCAGATCGAAGTGATCCGAGCAGCGACCAAGTCCATCGAGAGGGAGATAAATTCAGTCAACGACAACCCTCTGATTGATGTCTCGAGGAACAAGGCCATCCATGGAGGCAACTTCCAAGGGACCCCCATTGGCGTTTCCATGGACAACACCAGGCTCGCCATTGCTGCCATCGGGAAGCTCATGTTCGCCCAAATATCAGAGCTTGTCAATGACTTTTATAACAACGGCTTGCCTTCAAATCTATCCGGTGGGAGAAACCCTAGCTTGGATTATGGCTTCAAAGGCGCGGAGATAGCCATGGCTTCCTACTGCTCCGAGCTCCAGTACCTCGCCAATCCGGTCACAAACCATGTGCAGAGCGCCGAGCAGCACAACCAGGACGTGAACTCCCTGGGACTGATATCTTCGAGGAAGACGGGGGAGGCGGTGGAGATACTAAAGCTCATGACCTCCACCTTCCTGGTTGCACTCTGCCAAGCCATAGACTTGAGGCATCTGGAGGAGAACTTGAAGTGTGCCGTGAAGAATGCGGTGAGCCTGGCGGCAAAGAGGACTCTCACTTTCGGGGCCAATGGAGATCTTCATCCATCCAGGTTCTGCGAGAAGGATTTGATCAAGGTGGTAGATAAGGAGTATGTGTTCGCCTACGCCGACGATCCCTGCAGCTCTACCTACCCTTTGATGCAGAAGCTCAGGCAGGTGCTGGTTGAGCATGCCCTCAGCAACGGCGACAAGGAGAAGGCCAGGAGCACCTCCATCTTCCAAAAGATCACAGATTTTGAGGAGGATATCAATGCCGCGCTTCCCAAAGCGGTCGAGGCCGCCAGAGCGGCGTTTGAGAAGGGGTCGTCGGCGATAGAGAACAGAATCAAAGAATGCAGATCCTACCCACTGTACAGGCTTGTGAGGGAAGAGCTCGGGGCCGGCTTTCTCACCGGAGAGAAGGCGATGTCGCCAGGGGAGGAATTCGACAAGGTCTTCAATGCCATTTGCGAGGGGAGGGCGATAGATCCTCTGCTCGAGTGCTTGAAGGAGTGGAATGAAGCTCCTCTCCCTATTTGCTAG

> AfPAL [*Anoectochilus formosanus*] the sequence of phenylalanine ammonia-lyase protein from *Anoectochilus formosanus*

MDHARENGHVMENGHVTENGLCLKGKDPLGWIAAAKAVEGSHLEEVKRMVEDFRRPVVRLEGAELKISQVAAVAAGVVSQVQLAESARAGVNASSDWVMESMSAGGDHYGVTTGFGATSHRRTKQGGALQKELIRFLNAGIFGSGTNNTLPSAASRAAMLVRINTLLQGYSGIRFEILEAITSLLNSKITPCLPLRGTITASGDLVPLSYIAGVLTGRPNCKAITADGVTVNAVEAFRLAGISSGFFDLQPKEGLALVNGTAVGSGFASIVLFEANILALMAEVLSALFCEVMQGKPEFTDHLTHKLKHHPGQIEAAAIMEHVLEGSSYMKMAKKLHDLDPLQKPKQDRYALRTSPQWLGPQIEVIRAATKSIEREINSVNDNPLIDVSRNKAIHGGNFQGTPIGVSMDNTRLAIAAIGKLMFAQISELVNDFYNNGLPSNLSGGRNPSLDYGFKGAEIAMASYCSELQYLANPVTNHVQSAEQHNQDVNSLGLISSRKTGEAVEILKLMTSTFLVALCQAIDLRHLEENLKCAVKNAVSLAAKRTLTFGANGDLHPSRFCEKDLIKVVDKEYVFAYADDPCSSTYPLMQKLRQVLVEHALSNGDKEKARSTSIFQKITDFEEDINAALPKAVEAARAAFEKGSSAIENRIKECRSYPLYRLVREELGAGFLTGEKAMSPGEEFDKVFNAICEGRAIDPLLECLKEWNEAPLPIC-

>*ArPAL* [*Anoectochilus roxburghii*] the sequence of phenylalanine ammonia-lyase gene from *Anoectochilus roxburghii*

ATGGACCATGCTAGGGAGAACGGTCACGTGACGGAGAACGGGCACGTGACGGAGAACGGGCTATGCCTAAAGGGGAAGGACCCGCTGGGATGGATCGCGGCGGCGAAGGCGGTGGAGGGGAGCCACCTTGAGGAGGTGAAGCGGATGGTGGAGGACTTCCGGCGGCCGGTGGTGAGGCTCGAAGGAGCGGAGCTCAAAATATCGCAGGTAGCCGCAGTGGCTGCCGGCGTTGTTTCCCAAGTACAGCTAGCGGAGTCTGCGCGCGCTGGGGTGAATGCCAGTAGTGACTGGGTGATGGAGAGCATGAGTGCTGGTGGCGACCACTACGGCGTCACTACCGGCTTCGGCGCTACATCTCACCGCCGCACCAAGCAGGGCGGCGCCCTGCAGAAAGAACTCATCAGATTCCTTAATGCGGGGATCTTCGGATCAGGGACGAACAACACGCTGCCTTCGACCGCCAGCAGGGCTGCGATGCTTGTGAGGATCAACACCCTCCTCCAAGGTTACTCCGGCATCCGTTTTGAAATCCTGGAGGCCATTACCAGCCTCCTCAACAGCAAGATTACGCCTTGCCTGCCGCTGAGGGGAACCATCACCGCCTCCGGCGATCTTGTTCCTCTATCTTACATTGCGGGTGTCTTAACCGGCCGTCCCAATTGCAAGGCTATAACGGCCGACGGTGTTACTGTCAACGCAGTAGAGGCCTTCCGTCTTGCAGGAATCTCCAGCGGGTTCTTCGATCTTCAGCCCAAGGAAGGGCTCGCACTTGTCAATGGAACCGCCGTCGGCTCCGGCTTCGCCTCCATTGTCCTGTTCGAGGCAAACATCCTCGCCGTTATGGCAGAGGTTCTCTCTGCTCTGTTCTGCGAGGTGATGCAGGGGAAGCCGGAGTTCATCGACCACCTCACCCACAAGCTGAAACACCACCCAGGACAAATCGAGGCCGCCGCCATCATGGAGCACGTGCTTGAAGGAAGCTCCTACATGAAGATGGCCAAGAAGCTCCACGATTTGGATCCTCTTCAGAAGCCAAAGCAGGATCGCTATGCTCTCCGCACATCACCCCAATGGCTCGGCCCTCAGATCGAAGTGATCCGAGCGGCGACTAAGTCCATCGAGAGGGAGATCAATTCAGTCAACGACAACCCTCTGATTGATGTCTCGAGGAACAAGGCCATCCATGGAGGCAACTTCCAAGGGACCCCCATTGGCGTTTCCATGGACAACACCAGGCTCGCCATTGCTGCCATCGGGAAGCTCATGTTCGCCCAAATATCAGAGCTTGTCAATGACTTTTATAACAACGGCTTGCCTTCAAATCTATCCGGCGGGAGAAACCCGAGCTTGGATTATGGCTTCAAAGGCGCGGAGATAGCCATGGCTTCCTACTGCTCCGAGCTCCAGTACCTCGCCAATCCGGTCACAAACCATGTGCAGAGCGCCGAGCAGCACAACCAGGACGTGAACTCCCTGGGTCTGATATCTTCGAGGAAGACGGGGGAGGCGGTGGAGATACTAAAGCTCATGACCTCCACCTTCCTGGTTGCACTCTGCCAAGCCATAGACTTGAGGCATCTGGAGGAGAACTTGAAGTGTGCCGTGAAGAACGCTGTAAGCCTGGCGGCAAAGAGGACGCTCACTTTCGGGGCCAATGGAGATCTTCATCCATCCAGGTTCTGCGAGAAGGATTTGATCAAGGTGGTAGATAAGGAGTATGTGTTTGCCTACGCCGACGATCCCTGCAGCTCTACCTACCCTTTGATGCAGAAGCTCAGGCAGGTGCTGGTTGAGCATGCCCTCAGCAACGGCGACAAGGAGAAGGCCAGGAGCACCTCCATCTTCCAAAAGATCACAGATTTTGAGGAGGATATCAAAGCCGCGCTTCCCAAAGCGGTGGAGGCCGCCAGAGCGGCGTTTGAGAAGGGTTCGTCGGCCATAGAGAACAGAATCAAAGAATGCAGATCCTACCCACTGTACAGGCTTGTGAGGGAAGAGCTCGGCGCCGGATTTCTCACCGGAGAGAAGGCGATGTCGCCAGGGGAGGAATTCGACAAGGTCTTCAACGCCATTTGCGAGGGGAAGGCGATAGATCCTCTGCTCGAGTGCTTGAAGGAGTGGAATGAAGCTCCTCTCCCTATTTGCTAG

> ArPAL [*Anoectochilus roxburghii*] the sequence of phenylalanine ammonia-lyase protein from *Anoectochilus roxburghii*

MDHARENGHVTENGHVTENGLCLKGKDPLGWIAAAKAVEGSHLEEVKRMVEDFRRPVVRLEGAELKISQVAAVAAGVVSQVQLAESARAGVNASSDWVMESMSAGGDHYGVTTGFGATSHRRTKQGGALQKELIRFLNAGIFGSGTNNTLPSTASRAAMLVRINTLLQGYSGIRFEILEAITSLLNSKITPCLPLRGTITASGDLVPLSYIAGVLTGRPNCKAITADGVTVNAVEAFRLAGISSGFFDLQPKEGLALVNGTAVGSGFASIVLFEANILAVMAEVLSALFCEVMQGKPEFIDHLTHKLKHHPGQIEAAAIMEHVLEGSSYMKMAKKLHDLDPLQKPKQDRYALRTSPQWLGPQIEVIRAATKSIEREINSVNDNPLIDVSRNKAIHGGNFQGTPIGVSMDNTRLAIAAIGKLMFAQISELVNDFYNNGLPSNLSGGRNPSLDYGFKGAEIAMASYCSELQYLANPVTNHVQSAEQHNQDVNSLGLISSRKTGEAVEILKLMTSTFLVALCQAIDLRHLEENLKCAVKNAVSLAAKRTLTFGANGDLHPSRFCEKDLIKVVDKEYVFAYADDPCSSTYPLMQKLRQVLVEHALSNGDKEKARSTSIFQKITDFEEDIKAALPKAVEAARAAFEKGSSAIENRIKECRSYPLYRLVREELGAGFLTGEKAMSPGEEFDKVFNAICEGKAIDPLLECLKEWNEAPLPIC-
